# Randomized comparison of sevoflurane versus propofol-remifentanil on the cardioprotective effects in elderly patients with coronary heart disease

**DOI:** 10.1186/s12871-017-0397-0

**Published:** 2017-08-11

**Authors:** Yunlong Zhang, Wendong Lin, Sheliang Shen, Hongfa Wang, Xiaona Feng, Jiehao Sun

**Affiliations:** 1 Department of Anesthesiology, Zhejiang Provincial People’s Hospital, people’s hospital of Hangzhou Medical College, Hangzhou, China; 2 Department of Anesthesiology, 1st affiliated hospital of Wenzhou Medical University, 1#, nanbaixiang, Ouhai District, Wenzhou, 325000 China; 3 Department of Anesthesiology, Hangzhou 1st people’s hospital, Hangzhou, China

**Keywords:** Sevoflurane, Myocardial injury, cTnI, BNP

## Abstract

**Background:**

It is skeptical about cardioprotective property of sevoflurane in patients undergoing noncardiac surgery, especially in the elderly patients with coronary heart disease. We hypothesized that long duration of sevoflurane inhalation in noncardiac surgery could ameliorate myocardial damage in such patients.

**Methods:**

This was a randomized, prospective study. One hundred twenty-one elderly patients with coronary heart disease were randomly allocated into two groups. Maintenance of anesthesia was achieved by sevoflurane inhalation (Group S) or propofol-remifentanil respectively (Group PR). Serum cardiac troponin I (cTnI) and brain natriuretic peptide (BNP) were measured before anesthesia induction (T0), 8 h (T1) and 24 h (T2) after anesthesia respectively. The perioperative cardiac output, complications and postoperative 3-month follow-up from end of surgery were recorded.

**Results:**

Between the two groups, there were no statistical differences in the values of cTnI and BNP during the study. However, The area under the curve of cTnI values over 24 h after operation was less in Group S. Group PR had lower cardiac output and consumed more amount of phenylephrine during the study (*P* < 0.05).

**Conclusions:**

Compared with the group PR, sevoflurane had no benefit in the myocardial protection for the elderly patients with CHD. However, Sevoflurane showed advantage in maintaining hemodynamic stability during the operative period.

**Trial registration:**

Chinese Clinical Trial Registry, ChiCTR-IPR-16008871, 21 July 2016.

## Background

Myocardial injury can lead to more perioperative complications and prolonged hospitalization, especially for the elderly patients with coronary heart disease (CHD) undergoing major surgery. Perioperative myocardial damage occurs frequently in patients undergoing elective noncardiac surgery [1]. Many experimental studies have focused on the myocardial protection by sevoflurane in vivo and in vitro [2–6]. Sevoflurane was proved to decrease the size of the myocardial infarction before [4–6]. In contrast to experimental studies, clinical trials have shown inconsistent evidence regarding cardaic protection by sevoflurane. It was demonstrated that sevoflurane had the trend of myocardial protection in cardiac surgery [7]. However, meta-analysis [8] found that sevoflurane had no obvious myocardial protective effect in noncardiac surgery. In addition, sevoflurane may have age-associated bias in myocardial protection. Sevoflurane was observed to have myocardial protection only in young rats [9]. Robot-assisted or laparoscopic surgery, which has character of minimal surgical stressor (compared to large and open surgery), is more and more popular now. It is unclear whether volatile anesthetics in minimally invasive laparoscopic procedures could provide cardiac protection in CHD patients during the operations.

To the best of our knowledge, few clinical trials have confirmed the myocardial protection by long duration of sevoflurane inhalation in elderly patients with CHD before. In the present study, we hypothesized that long duration of sevoflurane inhalation in noncardiac surgery could ameliorate myocardial damage and stabilize hemodynamic responses in the elderly patients with CHD who are more susceptible to stress.

## Methods

### Patients involved in the trial

The study registered in Chinese Clinical Trial Registry (ChiCTR-IPR-16008871) was approved by the Institutional Research Ethics Committee of Zhejiang Provincial People’s Hospital (Protocol Number: 2104KY060, 2014–09-12). All of the patients involved in the research study have given their written informed consent for participation. The inclusion criterion of the clinical trial was elderly patients (65–80 years, ASA: II or III) scheduled for laparoscopic abdominal surgery, including pancreaticoduodenectomy and radical cystectomy, with an expected duration of operation ≥4 h. All patients were diagnosed with CHD by cardiac catheterization and corrected by stent implantation before. The serum level of cardiac troponin I (cTnI) and brain natriuretic peptide (BNP) were within the normal range, cardiac function status were NYHA I - II. Subjects were excluded from this study if the duration of operation was less than 4 h, or if the surgical procedure had to be changed to laparotomy, or surgical bleeding over 1000 ml. Other exclusion criteria were: if the patient was transferred to intense care unit after surgery, myocardial infarction in the last six months, hepatic or renal insufficiency, and a history of cerebral stroke, mental illness or preoperative hormone use. The trial is registered at http://www.chictr.org.cn/showproj.aspx?proj=15003.

### Protocol

Doctor Sun, Zhang, Lin and Wang enrolled the participants. A randomization code (Microsoft Excel 2013 generated by doctor Sun) was generated to assign patients (by doctor Zhang and Sun) into the two groups: Sevoflurane anesthesia group (group S), Propofol – Remifentanil intravenous anesthesia group (group PR). The allocation sequence was placed in sealed envelopes for each case, which was opened before each induction of anesthesia.

Upon hospitalization, patients were instructed by the anesthesiologist on the use of Numeric Rating Scale of Pain intensity – Visually (NRS) for assessing pain (0 cm = no pain, 10 cm = worst pain ever). After arrival in the operating theatre, standard cardiovascular and respiratory monitoring was established including heart rate, pulse oximetry, and invasive arterial pressure with a Datex-Ohmeda AS/3 monitor (GE Healthcare, Helsinki, Finland). Cardiac output (CO) and stroke volume variation (SVV) were recorded by FloTrac/Vigileo system (Edwards Lifesciences LLC). After preoxygenation, anesthesia induction was performed with sevoflurane inhalation (6–8 vol%) through mask with gas flow of 8 L/min until loss of consciousness and maintained in 3 vol% for 3 min in group S, or target-controlled infusion of propofol 3-4 μg/ml, remifentanil 4-6 ng/ml for the patients in group PR. Then 0.8 mg/kg rocuronium was given for tracheal intubation in all of the cases in the trial. In group S, patients were maintained with only inhalation of sevoflurane (end-expiratory concentrations of 0.5–2.0 MAC) without any other narcotics. In group PR, patients were maintained with target-controlled infusion of propofol 1–4 μg/ml and remifentanil 2–8 ng/ml. Anesthetic depth and cardiac output were adjusted according to changes of hemodynamic parameters and entropy index values (targeted range: 40–60). Hypotension/hypertension was corrected by adjusting the dose of anesthetics firstly within the acceptable anesthetic range. If it didn’t work, the change in hemodynamics should then be corrected by administering vasopressors/dilators. Urapidil 10–20 mg or phenylephrine 50–100 μg was given intravenously if the variation of the blood pressure was above 20% of the baseline. Moderate muscle relaxation, stable blood gas outcome, and urine output ≥1 ml/kg/h were achieved during the operation. Esophagus temperature were maintained at 36–37 degrees Celsius by using warming blanket (BearHuger, 3 M) and fluid warmer. The amount of fluid or blood transfusion was controlled based on surgical bleeding and SVV. Atropine was given for Bradycardia (heart rate < 45 /min). All patients had a patient controlled intravenous analgesia pump (sufentanil 2 μg/kg, 48 h) after surgery. The loading dose of sufentanil (10μg) was administered 20 min before the end of the surgery. The anesthesiologists who performed the anesthesia for the patients were not blinded to the allocation and not included in the postoperative assessment. Postoperatively, the episode of NRS_rest_ > 3 or NRS_sitting_ > 4 was documented according to the complaint from patients. Participants and anesthesiologists (Dr. Lin and Shen) who recorded the perioperative events were blinded to the allocation.

Dynamic monitoring of CO, SVV and ST-T change were recorded during the operation. The duration of surgery and anesthesia, vasoactive drug use and blood loss of the two groups were compared. Perioperative complications and postoperative 3-month follow-up from end of surgery were recorded. Venous blood for cTnI and BNP measurement was extracted at the following three time points: immediately before anesthesia induction (T0), 8 h (T1), and 24 h after anesthesia induction (T2) respectively.

Using the trapezium method, the area under the curve (AUC) of the cTnI value over the 24 h was calculated for AUC_cTnI-24 h_. To eliminate the influence of baseline value of cTnI, we calculated the new AUC (AUC_cTnI-subtracted_) from the subtracted cTnI value (cTnI_6h_-cTnI_baseline_, cTnI_24h_-cTnI_baseline_) which have subtracted the initial value of cTnI at baseline.

The primary outcome was the cTnI value on 8 h and 24 h after anesthesia induction (T1 and T2). All other outcomes were considered for secondary outcomes.

### Statistical analysis

Statistical analysis was performed with SPSS 13.0 for Windows (SPSS Inc., Chicago, IL, USA). Normal distribution of data was tested by the Kolmogorov–Smirnov test. The values of continuous data were analyzed using the ANOVA test (in case of normal distribution) or Mann-Whitney test (in case of non-normal distribution). Categorical data was assessed by the Χ^2^ test or Fisher’s exact test as appropriate. Time course data for BNP and cTnI values were performed by repeated measures ANOVA. The differences of results were considered as statistically significant if *P* < 0.05.

It was demonstrated that 40% and 10% cases in the TIVA and volatile anesthetics group respectively were found to have a detectable cTnI release [10, 11]. The authors calculated that a sample size of 59 patients per group would achieve 85% power to detect the difference of the values of cTnI between the two groups using one-way ANOVA (α = 0.05). To account for drop-outs, we recruited 65 patients to each group. Sample size estimates were done using PASS software (PASS 2008, Kaysville, UT, USA). Statistical analyses were done using SPSS 13.0 software (SPSS Inc., Chicago, IL, USA).

## Results

**Fig. 1:**
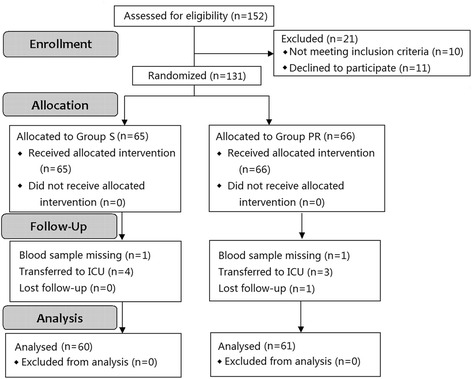
Flow of participants through the study

One hundred thirty-one subjects undergoing laparoscopic surgery were enrolled for the study from June 2014 to June 2016 in Zhejiang Provincial People’s Hospital and the 1st affiliated hospital of Wenzhou Medical University. Ten patients were excluded during the trial (Fig. 1 for CONSORT flow diagram). One hunderd twenty-one patients (60 in the Sevoflurane Group, 61 in the propofol-remifentanil Group) were included in the data analysis for our primary outcome. Groups were similar with respect to age, sex, BMI, physical status, recovery time, duration of surgery and anesthesia except the type of surgery (Table 1).

**Table 1:** Characteristics of patients

	S (*n* = 60)	PR (*n* = 61)	*P* value
Age (years)	71.83 ± 3.56	71.95 ± 3.96	0.373
BMI(kg/m2)	23.26 ± 1.29	23.31 ± 1.18	0.286
Male / Female	12 / 48	15 / 46	0.544
ASA (II/III)	46/14	42/19	0.355
Pathological coronary artery (1/2/3)	33/26/1	38/21/2	0.769
Smoke	36(60.0)	28(45.9)	0.082
History of diabetes	48(80.0)	42(68.9)	0.160
Hypercholesterolemia	50(83.3)	46(75.4)	0.282
Duration of surgery (min)	300 [290,340]	300 [290,320]	0.050
Duration of anesthesia (min)	330 [320,380]	340 [320,350]	0.407
Recovery time (min)	13[11,16]	12[11,15]	0.445
Type of surgery			
Pancreaticoduodenectomy	18(30.0)	31(50.8)	0.020
Radical cystectomy	42(70.0)	30(49.2)	0.020
Preoperative medication			
Statins	57(95.0)	53(86.9)	0.121
β-blockers	17(28.3)	12(20.0)	0.264
ACEI	18(30.0)	13(21.3)	0.274
CCB	42(70.0)	45(73.8)	0.645
Insulin	46(76.7)	43(70.5)	0.441
nitrates	0(0)	2(3.3)	0.252

*BMI* Body mass index, *CCB* calcium channel blocker, *ACEI* angiotensin-converting enzyme inhibitor

Values were expressed as number (percentage), mean ± SD or median [interquartile ranges]

Pathological coronary artery: the number of the diseased arteriosclerotic coronary artery vessels

**Fig. 2:**
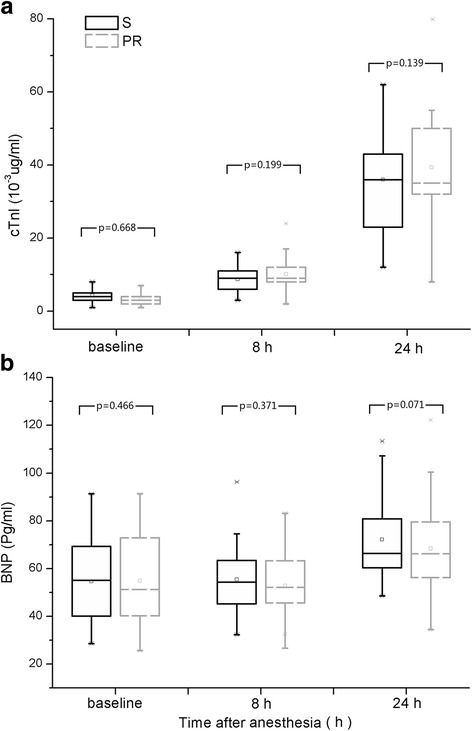
Box plots of cTnI and BNP values during the trial. (**a**) cTnI values during the trial (**b**) BNP values during the trial. Values are presented as median (square mark) with 25th to 75th percentiles (box), mean (horizontal bar in the box), 10th to 90th percentiles (whiskers) and upper, low limit (star shape)

cTnI/BNP (upper/lower panel) at the different times of measurement are shown in Fig. 2. There was no significant difference between groups for any of these variables. AUC of cTnI release at 24 h are displayed in Table 2. AUC_cTnI-24 h_ of group S was found to be less than that of group PR. Even after we eliminate the influence of the baseline value of cTnI, AUC_cTnI-subtracted_ of group S was still less than that of group PR (Table 2).

**Table 2:** Characteristics of hemodynamic drugs used in operation, side effect and postoperative follow-up

	S (*n* = 60)	PR (*n* = 61)	*P* value
AUC_cTnI-24 h_ (μg/ml)	0.408[0.364,0.456]	0.456[0.416,0.496]	0.001
AUC_cTnI-subtracted_(μg/ml)	0.312[0.244,0.324]	0.376[0.324,0.412]	0.001
Estimated blood loss(ml)	520[430/587.5]	460[395/570]	0.015
Number of blood transfusion	11(18.3)	10(16.4)	0.778
Total blood transfusion(ml)	200[200,200]	200[200,300]	0.122
Urapidil(mg)	30[0/40]	20[0/40]	0.621
Phenylephrine(μg)	300[200/400]	600[400/800]	0.001
Side effect			
Delirium	5(8.3)	2(3.3)	0.213
Nausea	15(25.0)	12(20.0)	0.482
Complaint of pain after operation	12(20.0)	15(24.6)	0.544
Postoperative 3-month follow-up			
Myocardial ischemia^a^	2(3.3)	3(5.0)	0.508
Supraventricular tachycardia	0(0)	1(1.7)	0.504
All-cause mortality	1(1.7)	0(0)	0.496

*AUC* area under the curve

Values were expressed as number (percentage) or median [interquartile ranges]

^a^Myocardial ischemia: diagnosis by ECG and cTnI

Both in group S and group PR, the values of cTnI were significantly increased at T1 and T2 compared with that of T0. Compared with the cTnI value at T0 in group S, the values increased at T1 (*p* = 0.001, Z = −7.994) and T2 (*p* = 0.001, Z = −9.469) respectively. In group PR, the values of cTnI were also increased at T1 (*p* = 0.001, Z = −8.602) and T2 (*p* = 0.001, Z = −9.548) compared with at T0, respectively. However, we detected no such increment of BNP values at the three time points in both groups (Fig. 2).

**Fig. 3:**
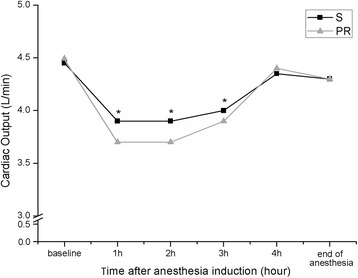
Cardiac output after anesthesia induction. S: Group sevoflurane. PR: Group propofol and remifentanil. Cardiac output are presented as Median. *p < 0.01 versus Group PR

The hemodynamic status was as shown in Fig. 3. Compared with group PR, cardiac output at 1 h (*p* = 0.001, Z = −24.236), 2 h (*p* = 0.001, Z = −19.292), 3 h (*p* = 0.001, Z = −14.994) after induction in group S was higher. Meanwhile, group PR consumed more phenylephrine during the operation (Table 2). There was no obvious ST-T elevation or depression in any involved case.

Perioperative complications and postoperative 3-month follow-up from end of surgery were given in Table 2. One case in group PR suffered from supraventricular tachycardia needing hospitalization. One case in group S died 2 months later due to multiple organ dysfunctions.

## Discussion

Volatile anesthetic was demonstrated to provide protective effect against ischemic myocardial damage [2–6]. However, in a recent meta-analysis [12], volatile anesthetic was only found to reduce mortality and perioperative complications in cardiac, but not in noncardiac, surgery. Our findings were in line with the conclusion in the meta-analysis, where the use of volatile anesthetic was not associated with reduced mortality or lower incidences of pulmonary and other complications in noncardiac surgery compared to total intravenous anesthesia.

Elderly patients with CHD are sensitive to stress response and prone to cardiac injury, which is characterized by increment of cTnI values after surgery. Sevoflurane is a widely used inhalation anesthetic with rapid onset and recovery property, which enable quick regulation of the depth of anesthesia. The myocardial protective property of sevoflurane have been reported by many studies [5–7]. Compared with propofol, sevoflurane could also shorten the hospital length in cardiac surgery [13].

However, a reduction in myocardial ischemia by sevoflurane has not been detected in noncardiac surgery [14]. Compared with the values of cTnI and BNP in group PR, group S had no benefit in reducing values of cTnI or BNP at any time point observed during the trial. Long duration (more than 4 h) of sevoflurane inhalation did not provide clinically detectable myocardial protection in elderly patients with CHD in this study. These results are similar to the previous studies [15, 16]. RCT which included 385 cases in Switzerland found that, propofol and sevoflurane had no difference in postoperative delirium, myocardial ischemia, and major adverse cardiac events during 1 year follow-up [15].

Interestingly, the AUC of the cTnI over 24 h after operation in group S was found to have less value compared with group PR. Did it mean that sevoflurane has more benefit in myocardial protective effect? In previous study [13], it was demonstrated that the AUC of cTnI values during the trial was similar between sevoflurane and propofol in cardiac surgery. In the current trial, it may have detected the trend of myocardial protection by sevoflurane. It still needs further studies to prove the trend. Is the trial inadequately powered to determine the difference between the two groups? Most of the similar trials failed to detect the differences even with more samples. Based on some hypothesized effect [17], the recommended sample size using PASS software to assess a possible effect of sevoflurane on myocardial ischemia incidence would amount to >3000 patients, or even >10,000 patients.

Hypercholesterolemic myocardium was vulnerable to ischemia-reperfusion injury and refractory to sevoflurane-induced protection [18]. Both of the two groups in the trial had no difference in the history of hypercholesterolemia.

The cardiac effects of sevoflurane can be different in the elderly population. Compared with younger patients, Nakao [19] found that sevoflurane had a much greater risk of QT interval prolongation. The use of sevoflurane in elderly patients susceptible to ventricular arrhythmias could markedly increase the potential for serious arrhythmias. Sevoflurane could strongly suppress cardiac effect when used in high concentration [20] and hence induce myocardial toxicity. So we modulated the concentration of sevoflurane between 0.5–2.0 MAC to avoid such cardiac toxicity in our study.

The incidence of myocardial ischemia by sevoflurane from the trial of Lurati was more than 40%, much higher than that in this trial [15]. Maybe the criterion in the two studies was different. The severe cases transferred to ICU, were excluded from the analysis in the trial. That might be the reason that we had lower incidence of myocardial ischemia.

Studies have shown that the duration of hypotension in the operation is significantly associated with the degree of myocardial ischemia [21, 22]. For the elderly patient with CHD, the cardiac functional reserve is limited, and the hemodynamic status tends to be unstable. In order to explore the impact on myocardial parameters by long duration of sevoflurane inhalation, we ruled out the influence of hypotension induced ischemia, hypoxia, hypercapnia, massive surgical bleeding and other stress which could lead to myocardial injury. We deliberately kept SpO_2_ over 98%, normocapnia and the fluctuations of blood pressure less than 20% of the base value during the operation. The negative finding with no myocardial protection by sevoflurane from this trial did not mean that sevoflurane could not provide such protection in CHD patients. Instead, it mean that patients outcome could be affected by many anesthesia-related or surgery-related factors.

Although sevoflurane had no benefit in perioperative cardiac protection in comparison with group PR in this trial., propofol caused more hypotension than sevoflurane in elderly patients with CHD in this trial. It was not surprising that the propofol group had lower cardiac output so as to cause hypotension and reflex tachycardia .

Myocardial injury markers include CK-MB, cTnI, C-reactive protein and BNP, et al. The extent of cTnI elevation is associated with the magnitude of myocardial damage [23]. When myocardial injury occurred, cTnI was quickly released into the peripheral blood circulation, and peaked at about 24 h. So we chose the time points of 8 h and 24 h after induction to test the value of cTnI.

BNP, a quantitative marker of heart failure, could reflect the ventricular systolic and diastolic dysfunction, as well as the severity by acute hemodynamic change. It is known that the possibility of heart failure is up to 95% when the serum level of BNP is over 400 pg/ml [24]. None of the elderly patients have experienced the myocardial dysfunction in this trial. All of the BNP values in the cases were less than 100 pg/ml.

In the present study, the values of cTnI had significantly increased at T1 and T2 compared with that of T0, which suggested that the long duration of surgery could induce myocardial injury, especially in the elderly patients who had CHD undergoing major surgery. Strict monitoring and management of hemodynamic fluctuation are more important than choosing the type of anesthetic to prevent myocardial injury for the elderly patients with CHD undergoing noncardiac surgery.

### Limitation

Sevoflurane was proved to have effect of myocardial protection compared with normal saline in animals before [5]. Due to the ethical reasons, sevoflurane could not be designed to prove the advantage in inhibiting myocardial injury compared with normal saline in human. To minimize the interference of other drugs, no other narcotic was administered in the sevoflurane group. Even under this conditions, the administration of sevoflurane was not associated with clinically relevant benefit. Actually propofol [25] plus remifentanil [26] also might have myocardial protective effect. Although we did not conclude that sevoflurane had no benefit in cardiac protection compared with propofol plus remifentanil, we also could not rule out the possibility of myocardial protective effect by sevoflurane. Preclinical studies have previously shown that the cardiac protective effect of sevoflurane is concentration dependent [27] which may be a cause of some of the negative findings here (since sevoflurane concentrations were given over a range of 0.5–2.0 MAC). Owing to clinical research here, it is impossible to fix the concentration of the sevoflurane inhalation without any other narcotics in the clinical trial.

## Conclusions

Compared with propofol-remifentanil intravenous anesthesia, long duration of sevoflurane inhalational anesthesia had no benefit on cardiac protection based on the examination of myocardial injury markers in the elderly patients with CHD in elective abdominal surgery. However, sevoflurane was more advantageous to maintain hemodynamic stability. Due to lack of cases, low mortality rate in modern surgery, as well as lots of interfering factors, large, multicenter studies are needed in the future trials to clarify the cardiac protective effect of sevoflurane on the elderly patients with CHD.

## Abbreviations

AUC: The area under the curve
CHD: Coronary heart disease
CO: Cardiac output
SVV: Stroke volume variation
